# Diagnostic performance of multimodal large language models in detecting pulp stones on panoramic radiographs

**DOI:** 10.1186/s12903-026-10119-6

**Published:** 2026-10-08

**Authors:** Bianca Marques de Mattos de Araujo, Maria Eduarda Cavassin, Allan Abuabara, Maria Luisa Giacon, Luiz Fernando Fariniuk, Christian Kirschneck, Michelle Nascimento Meger, Rafaela Scariot, Flares Baratto-Filho, Erika Calvano Küchler

**Affiliations:** 1 School of Dentistry, Tuiuti University of Paraná, Curitiba, Paraná Brazil; 2 University of the Region of Joinville (Univille), Joinville, Santa Catarina Brazil; 3 Department of Orthodontics, Medical Faculty, University Hospital Bonn, Welschnonnenstr. 17, Bonn, 53111 Germany; 4 Department of Stomatology, Federal University of Parana, Curitiba, Paraná Brazil

**Keywords:** Dental pulp calcification, Large language models, Artificial intelligence

## Abstract

**Background:**

Pulp stones are calcified structures within the dental pulp that may increase the complexity of endodontic treatment and are commonly identified through radiographic examinations. Despite recent advances in artificial intelligence applied to dental imaging, the diagnostic capability of publicly available multimodal large language models (LLMs) for detecting pulp stones under zero-shot conditions remains unclear. Therefore, this study evaluated the diagnostic performance and response consistency of multimodal LLMs in detecting pulp stones on panoramic radiographs of posterior teeth.

**Methods:**

This comparative and exploratory study evaluated the GPT-5 and Gemini 2.5 Flash multimodal LLMs using panoramic radiograph image crops containing posterior teeth. A total of 64 image crops, comprising 192 posterior teeth, were analyzed. The models were tested under standardized zero-shot conditions using independent sessions and a standardized English-language prompt. Each image was submitted six times to both models to assess response consistency. Diagnostic performance was evaluated using accuracy, sensitivity, specificity, positive predictive value, negative predictive value, and F1-score with 95% confidence intervals. Intra-model consistency was assessed using majority agreement, complete agreement across repetitions, and Fleiss’ Kappa coefficient.

**Results:**

The models demonstrated similar overall accuracy for binary detection of pulp stones, with 56.5% for ChatGPT and 57.0% for Gemini. ChatGPT showed high specificity (96.2%) and greater intra-model stability, whereas Gemini demonstrated higher sensitivity (81.8%) and superior performance in identifying the affected tooth and dental group. However, Gemini also presented lower specificity and greater variability across repeated evaluations. Fleiss’ Kappa coefficients indicated low intra-model agreement beyond chance for both models, particularly for Gemini.

**Conclusion:**

Multimodal LLMs demonstrated limited capability in detecting pulp stones on panoramic radiographs, with distinct diagnostic profiles between the evaluated models. Although the findings suggest potential future applications of LLMs as auxiliary tools for dental image interpretation, their current performance does not yet support consistent clinical applicability for pulp stone detection.

**Supplementary Information:**

The online version contains supplementary material available at https://doi.org/10.1186/s12903-026-10119-6.

## Background

Pulp calcifications, also known as pulp stones, are mineralized structures present within the dental pulp tissue and may be observed in both the coronal and radicular regions [1]. These formations may occur freely within the pulp chamber or attached to dentin, with a wide variation in prevalence, estimated to range from 8% to 95%, depending on individual factors and study methodologies [2]. Primarily associated with pulpal aging, these calcifications may coexist with physiological regressive changes, including reduced vascularization, decreased cellular density, and a fibrotic appearance of the pulp tissue [3, 4].

Some factors such as dental trauma and orthodontic treatment have also been associated with an increased frequency of pulp stones, suggesting that external factors such as local trauma and orthodontic forces may promote vascular alterations and calcification processes within the dental pulp [5–7]. From a clinical perspective, pulp stones represent a significant challenge in endodontic practice, as they may hinder or even prevent access to the root canals and apical foramen, compromising adequate instrumentation and disinfection of the root canal system. Consequently, the presence of pulp calcifications is directly associated with increased complexity of endodontic treatment, particularly in narrow or obliterated canals [8].

Despite their clinical relevance, the detection of pulp stones through radiographic examinations, particularly panoramic radiographs, still presents important limitations due to structural superimposition, lower spatial resolution, and variability in interpretation among examiners. In this context, the application of artificial intelligence–based tools has emerged as a promising strategy to assist in the analysis of dental images, contributing to greater standardization and diagnostic accuracy [9]. However, although deep learning models have been extensively investigated for the detection of dental abnormalities, the use of large language models (LLMs) in the interpretation of radiographic images remains limited [10, 11], particularly regarding the identification of pulp stones.

Unlike task-specific deep learning systems, which are developed and trained for a specific diagnostic purpose, multimodal LLMs are publicly available general-purpose models capable of analyzing images without domain-specific training. Their increasing accessibility has stimulated interest in their use for health-related information and decision support, despite not being specifically designed for healthcare applications [10–13]. Consequently, evaluating whether these models can identify subtle radiographic findings, such as pulp stones, and provide consistent responses across repeated evaluations is clinically relevant. Although previous studies have investigated multimodal LLMs in different dental imaging tasks [10, 11], evidence regarding their performance in pulp stone detection remains scarce.

Given the high prevalence of these alterations and the growing incorporation of artificial intelligence into dentistry, investigating the potential of LLMs, such as GPT-5 and Gemini 2.5 Flash, for the identification of pulp stones in imaging examinations is warranted. Therefore, the present study aimed to evaluate the diagnostic performance and response consistency of publicly available multimodal LLMs for detecting pulp stones on panoramic radiographs of posterior teeth under standardized zero-shot conditions.

## Methods

### Study design

This was a comparative and exploratory study designed to evaluate the performance of the GPT-5 (OpenAI) and Gemini 2.5 Flash (Google Inc.) LLMs in detecting pulp stones on panoramic radiographs. A convenience sample was used, consisting of images obtained from a previously existing radiographic database.

The study was approved by the Research Ethics Committee under protocol number 6.973.530 and conducted in accordance with the principles of the Declaration of Helsinki. All participants provided written informed consent. The images were anonymized before being accessed by the researchers between November 2025 and March 2026.

### Data source and sample selection

**Fig. 1:**
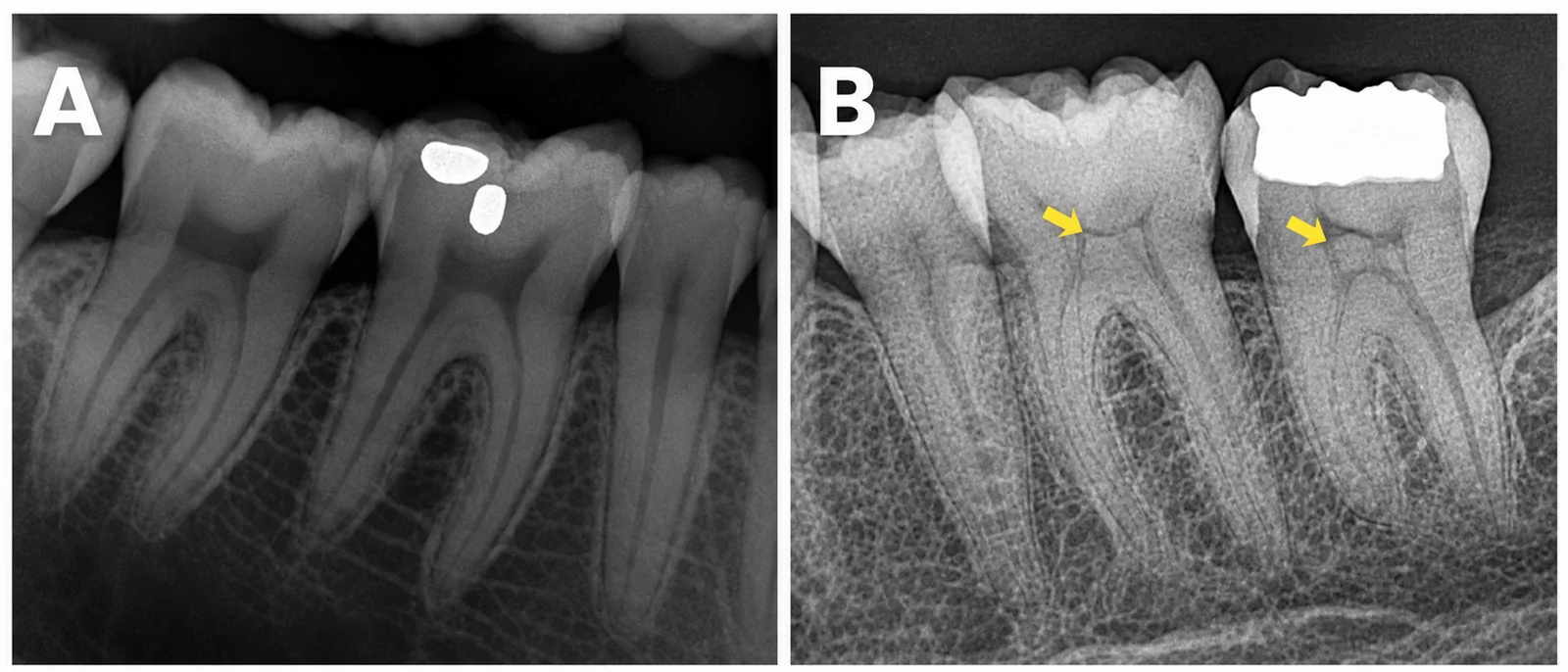
Representative standardized panoramic radiograph image crops used for model evaluation. **A** Negative image crop without radiographic evidence of pulp stones according to the reference standard established by the endodontic specialist. **B** Positive image crop showing pulp stones (arrows)

Images from adult patients of both sexes with complete root formation and the presence of posterior teeth in at least one quadrant were included. Radiographs presenting severe artifacts, major anatomical superimposition impairing visualization of the region of interest, image distortions, developmental anomalies affecting tooth morphology, or unsatisfactory image quality were excluded. All radiographs were obtained under standardized clinical conditions using a digital panoramic imaging system (Axeos; Dentsply Sirona, Frankfurt, Germany). For the evaluation of calcifications, the images were randomly selected with an exclusive focus on posterior teeth, including premolars and molars. To direct the analysis of the LLMs toward the regions of interest, standardized image crops containing groups of up to three adjacent posterior teeth were created, fully preserving the coronal portion, root apices, and adjacent alveolar bone (Fig. 1). No image enhancement, filtering, or additional preprocessing procedures were applied.

### Image classification

The images were classified according to two criteria: presence or absence of a pulp stone and identification of the affected tooth within the analyzed image crop. Pulp stones were defined as well-demarcated radiopaque structures located within the pulp chamber or root canal system. The classifications performed by the specialist were used as the reference standard for evaluating the performance of the language models.

All evaluations were conducted by an endodontic specialist with more than ten years of clinical experience under standardized digital viewing conditions. The examiner performed all analyses independently and without access to the responses generated by the language models. In cases of diagnostic uncertainty, the images were reassessed by the same examiner until a final classification was established.

To assess intraexaminer reproducibility, all images were reevaluated after a two-week interval. Agreement was determined using Cohen’s Kappa coefficient at the tooth level, considering all evaluated teeth with and without pulp stones. The kappa value was 1.00, indicating perfect diagnostic agreement.

### Sample size

Sample size calculation was performed assuming an infinite population, a 5% margin of error, a 95% confidence level, and an expected accuracy proportion of 50%. This conservative estimate was adopted due to the absence of previous data regarding the performance of the evaluated models for this outcome. The calculation indicated that 384 model inferences would be required for the planned evaluation. Because the study was designed to assess both diagnostic performance and response consistency, 64 panoramic radiograph image crops were selected and each image was submitted six times in independent sessions to both language models, generating 384 model inferences per model.

The final dataset consisted of 64 image crops with a nearly balanced distribution between cases with and without pulp stones, totaling 192 posterior teeth evaluated. The cropped radiographic images always focused on a central molar, including one adjacent tooth mesially and one distally.

### Prompt, LLM structure, and evaluation procedure

Two LLMs capable of simultaneously processing text and images were evaluated: GPT-5 (OpenAI; https://chat.openai.com/) and Gemini 2.5 Flash (Google Inc.; https://gemini.google.com/). Both models were accessed through their publicly available web interfaces using the free multimodal versions available between November 2025 and March 2026. The models were tested independently under standardized conditions using the default settings provided by each platform, without modification of internal parameters.

The prompts were developed in English using an objective and standardized format to ensure consistency of instructions between the models. In each test, a panoramic radiograph image crop containing up to three posterior teeth (maxillary or mandibular) was individually presented together with the following instruction:

Does this image show a pulp stone in any tooth? If yes, which tooth?

The images were presented in random order, and each inference was performed in a new independent session without access to previous interactions, thereby avoiding contextual memory influence between tests. The prompt formulation was designed to evaluate exclusively the visual interpretation capability of the models, without providing additional clinical context, prior examples, or auxiliary information.

The responses generated by the models were recorded according to the presence or absence of a pulp stone and, when applicable, identification of the affected tooth within the analyzed image crop. For localization analyses, responses were subsequently coded at the tooth level according to the teeth identified by the model. To assess response consistency, each image was submitted six times to both models in independent sessions. Subsequently, all responses were stored, coded, and compared with the reference standard established by the endodontic specialist to determine diagnostic agreement and errors.

All tests were conducted under zero-shot conditions, without fine-tuning, supervised training, or advanced prompt engineering, thereby preserving the original behavior of the models. This approach allowed the intrinsic diagnostic performance of the LLMs to be evaluated in a scenario closer to real-world use by non-specialized users while also ensuring direct comparability between the generated responses.

### Model performance analysis

The diagnostic performance of the models was evaluated using accuracy, sensitivity, specificity, positive predictive value (PPV), negative predictive value (NPV), and F1-score, all reported with 95% confidence intervals (95% CIs). The 95% confidence intervals for proportions were estimated using the Wilson method, whereas the 95% confidence intervals for the F1-score were estimated by bootstrap with 2,000 resamplings due to the non-binomial distribution of this composite metric.

Comparisons between ChatGPT and Gemini regarding binary classification accuracy, tooth identification accuracy, and dental group identification accuracy were performed using McNemar’s test, considering the paired nature of the responses. For McNemar analyses, each paired observation consisted of the responses generated by ChatGPT and Gemini for the same image and repetition, thereby preserving the paired structure of the comparison between models. In addition, Generalized Estimating Equations (GEE) models with binomial distribution and patient identifier as the clustering unit were applied to account for within-patient correlation arising from repeated model evaluations and the inclusion of multiple image crops from some participants.

Tooth identification was considered correct when the model indicated the same tooth as the reference standard using a dental numbering system. Model responses containing tooth numbers were coded according to the indicated tooth and compared with the reference standard. Localization analyses were performed at the tooth level. When multiple teeth were mentioned by the model, each reported tooth was coded and compared individually with the tooth-level reference standard. Therefore, inclusion of the reference tooth was counted as a correct localization for that tooth, whereas additional incorrectly identified teeth were recorded as localization errors in their respective tooth-level evaluations. Dental group identification was also evaluated and considered correct when the model indicated a homologous tooth within the same anatomical group, even if located on the contralateral side.

Intra-model consistency was assessed based on the six independent repetitions for each image. Mean majority agreement, the proportion of complete agreement across repetitions, and Fleiss’ Kappa coefficient were calculated. The 95% confidence intervals for the proportion of complete agreement were estimated using the Wilson method, whereas the 95% confidence intervals for Fleiss’ Kappa were estimated by bootstrap when feasible. In addition, a forest plot was created to graphically represent the diagnostic metrics and their respective 95% confidence intervals, allowing visual comparison of model performance.

All statistical analyses were performed in Python (version 3.10.12) using the Scikit-learn, Statsmodels, NumPy, and Pandas libraries.

## Results

The sample consisted of 64 panoramic radiograph image crops, totaling 192 posterior teeth evaluated in adult individuals. Among the analyzed image crops, 33 (51.6%) presented at least one pulp stone according to the reference specialist’s evaluation, whereas 31 (48.4%) showed no evidence of the alteration. A total of 35 teeth with pulp stones were identified within the 33 positive image crops. The distribution of teeth affected by pulp stones is presented in Table 1.

**Table 1:** Distribution of teeth affected by pulp stones

Dental arch	Tooth	Frequency *n* (%)
Maxillary	Right first molar	9 (25.7%)
	Right second molar	2 (5.7%)
	Right third molar	1 (2.9%)
	Left first molar	10 (28.6%)
	Left second molar	1 (2.9%)
	**Subtotal**	**23 (65.7%)**
Mandibular	Left first molar	3 (8.6%)
	Left second molar	4 (11.4%)
	Right first molar	4 (11.4%)
	Right second molar	1 (2.9%)
	**Subtotal**	**12 (34.3%)**

Frequencies refer to teeth affected by pulp stones. Since some image crops contained more than one affected tooth, 33 positive image crops corresponded to a total of 35 affected teeth

### LLM performance

**Fig. 2:**
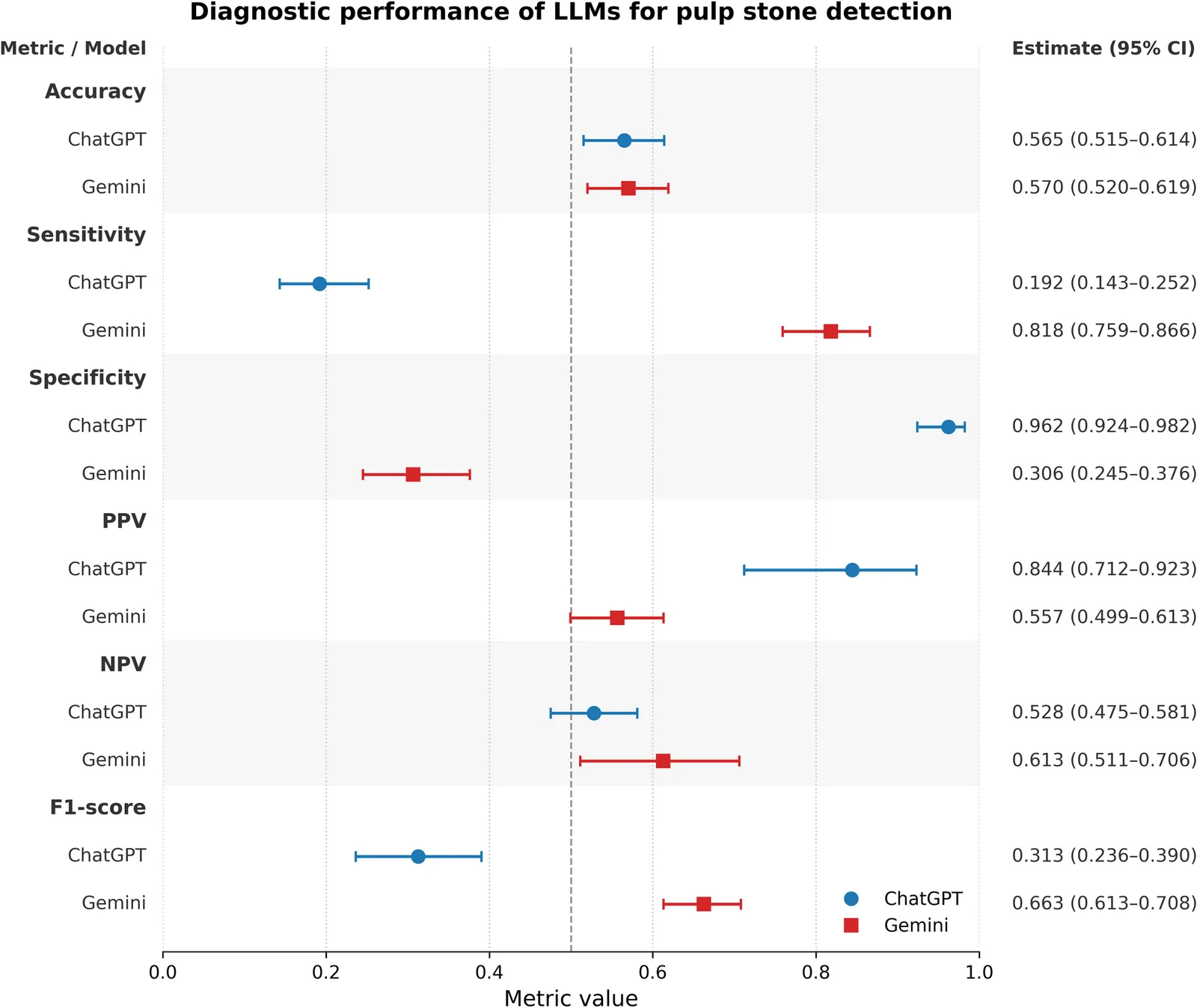
Forest plot of the diagnostic performance of the LLMs for pulp stone detection

The models demonstrated similar overall performance in the binary detection of pulp stones, with an accuracy of 56.5% for ChatGPT (95% CI: 51.5–61.4%) and 57.0% for Gemini (95% CI: 52.0–61.9%). Despite the similar overall accuracy, the models exhibited distinct diagnostic profiles. ChatGPT demonstrated high specificity (96.2%; 95% CI: 92.4–98.2%) and positive predictive value (84.4%; 95% CI: 71.2–92.3%), but low sensitivity (19.2%; 95% CI: 14.3–25.2%), indicating a greater tendency to correctly identify images without pulp stones, although with limited ability to detect positive cases. In contrast, Gemini demonstrated high sensitivity (81.8%; 95% CI: 75.9–86.6%) but low specificity (30.6%; 95% CI: 24.5–37.6%), indicating greater ability to detect images containing pulp stones, although with a high frequency of false-positive results. The F1-score values were 0.313 (95% CI: 0.236–0.390) for ChatGPT and 0.663 (95% CI: 0.613–0.708) for Gemini (Fig. 2). No significant difference was observed between the models regarding overall binary classification accuracy (*p* = 0.950). Confusion matrices at the repeated-inference level are provided in the Supplementary Material 1.

In the anatomical identification of pulp stones, Gemini demonstrated superior performance compared with ChatGPT. The proportion of correct identification of the affected tooth was 22.7% (87/384; 95% CI: 18.8–27.1%) for Gemini and 9.1% (35/384; 95% CI: 6.6–12.4%) for ChatGPT. A similar pattern was observed for dental group identification accuracy, considering errors limited to the contralateral side, with rates of 35.4% (136/384; 95% CI: 30.8–40.3%) for Gemini and 9.1% (35/384; 95% CI: 6.6–12.4%) for ChatGPT. These differences were statistically significant for both affected tooth identification (*p* < 0.001) and dental group identification (*p* < 0.001).

The analysis accounting for the dependency of repeated evaluations for each image confirmed the absence of a significant difference between the models regarding overall binary classification accuracy (*p* = 0.952). However, Gemini maintained significantly superior performance in identifying the affected tooth (β = 1.072; *p* < 0.001) and the dental group (β = 1.699; *p* < 0.001).

### Intra-model consistency

The intra-model consistency analysis demonstrated greater stability in the responses generated by ChatGPT across independent repetitions of the same images. For the detection of pulp stone presence, the model showed a mean majority agreement of 90.9%, with complete agreement across all repetitions in 62.5% of the images (95% CI: 50.25–73.33%). Similar results were observed for identification of the affected tooth, with a mean majority agreement of 89.8% and complete agreement in 62.5% of the images (95% CI: 50.25–73.33%), indicating high reproducibility of the responses generated by the model.

Gemini demonstrated lower stability across repeated evaluations of the same images. For pulp stone presence, the mean majority agreement was 77.9%, with complete agreement across all repetitions in 37.5% of the images (95% CI: 26.67–49.75%). In the identification of the affected tooth, response variability was even more pronounced, with a mean majority agreement of 42.7% and absence of complete agreement across all repetitions (0%; 95% CI: 0.00–5.66%), suggesting greater inconsistency in the anatomical localization of pulp stones.

Fleiss’ Kappa coefficient for pulp stone presence was 0.260 for ChatGPT and 0.112 for Gemini, indicating low intra-model agreement beyond chance for both models, particularly for Gemini.

## Discussion

The presence of pulp stones represents a challenge in dentistry due to their high prevalence, clinical relevance during endodontic procedures, and association with local and systemic factors. These calcifications are described as radiopaque structures located within the dental pulp and are frequently observed in posterior teeth, particularly maxillary molars [14, 15]. Although generally asymptomatic, pulp stones may hinder access to the root canals and compromise different stages of endodontic treatment [14]. In addition, LLMs have been investigated as auxiliary tools for the interpretation of dental radiographic images and support for clinical decision-making, particularly because of their multimodal capability to integrate text processing and image analysis [10, 11]. In this context, the present study explored the ability of publicly available multimodal LLMs to detect pulp stones and provide consistent responses across repeated evaluations of panoramic radiographs under zero-shot conditions. The results demonstrated relevant differences between the evaluated models. While ChatGPT showed high specificity, Gemini demonstrated greater sensitivity and superior performance in the anatomical identification of the affected tooth. Nevertheless, both models presented limitations regarding intra-model consistency and overall agreement of the responses.

Studies involving LLMs applied to Dentistry have demonstrated that, although diagnostic performance may vary according to the complexity of the radiographic alteration, the accuracy of these tools has remained relatively consistent regardless of the type of radiographic examination used. In dental and maxillofacial radiology, some multimodal models have already demonstrated diagnostic performance very close to that of human specialists in general interpretation tasks, in addition to exhibiting high intra-model reproducibility [16]. On the other hand, studies involving external apical root resorption have reported important limitations, including low agreement with specialists, high variability across repeated evaluations, and difficulties in classifying the severity of radiographic alterations [11]. In analyses involving periapical lesions, a tendency toward excessive positive classifications has also been observed, resulting in high sensitivity accompanied by low specificity and frequent errors in tooth localization [10]. The findings of the present study partially corroborate these observations, demonstrating that the performance of LLMs in detecting pulp stones varies according to the evaluated metric. While Gemini showed greater sensitivity and better anatomical localization of pulp stones, ChatGPT demonstrated higher specificity and greater intra-model stability. However, greater response stability should not be interpreted as superior diagnostic performance. Although ChatGPT showed higher majority agreement and complete agreement across repeated evaluations, it also presented very low sensitivity, indicating a tendency to repeatedly classify images as negative. Therefore, part of the observed stability may reflect a consistent response pattern rather than consistently correct detection of pulp stones. These findings highlight that response consistency and diagnostic correctness represent distinct characteristics and should be interpreted together when assessing the clinical applicability of multimodal LLMs.

Orthodontic forces may induce vascular and inflammatory changes capable of favoring pulpal calcification processes. Studies based on panoramic radiographs have demonstrated a significant increase in the frequency of pulp stones following orthodontic treatment, regardless of whether extractions were performed, with molars, particularly maxillary molars, being the most frequently affected teeth [5, 7, 17]. Panoramic radiography remains widely used in Orthodontics because it allows simultaneous evaluation of both dental arches and longitudinal patient follow-up, although it presents limitations related to anatomical superimposition and lower spatial resolution for identifying small calcifications [5, 7]. These limitations may affect both human interpretation and the performance of artificial intelligence–based tools, particularly in subtle alterations or those located in radicular regions. Similarly, studies using deep learning for the automatic detection of pulp stones on panoramic radiographs have also reported difficulties associated with the small size of calcifications, the presence of artifacts, and anatomical superimposition [18]. These factors may contribute to the differences in performance observed between the evaluated models, particularly in tasks requiring precise visual recognition and anatomical localization of subtle radiographic alterations.

Despite the recent advances in multimodal LLMs applied to medical image interpretation, studies have demonstrated that these models still present important limitations related to diagnostic consistency, visual recognition, and clinical reproducibility. Relevant differences in performance between models have been observed even in tasks involving simplified or standardized images, with frequent classification errors, low stability across repeated evaluations, and difficulty in interpreting subtle alterations or structures with high morphological complexity [19, 20]. Furthermore, studies in radiology and histopathology have demonstrated that the visual performance of these systems remains inferior to that observed in text-based tasks, suggesting limitations in their ability to process and recognize complex anatomical patterns [19, 20]. In applications involving radiographs, limitations related to diagnostic accuracy and clinical applicability have also been reported, even under controlled evaluation conditions [21, 22]. Similarly, studies involving dental age estimation using panoramic radiographs have demonstrated greater variability and lower reproducibility of LLMs when compared with traditional methods performed by human specialists, reinforcing the need for caution in the clinical interpretation of results generated by these systems [23]. These findings reinforce that, although multimodal LLMs show promising potential for applications involving medical imaging, the performance of these systems still depends on the type of task, the morphological complexity of the evaluated alterations, and the stability of the generated responses, factors that may currently limit their clinical applicability in the interpretation of dental radiographs.

The present study has some limitations that should be considered when interpreting the findings. First, only panoramic radiographs of posterior teeth were used, an imaging modality that presents inherent limitations related to spatial resolution and anatomical superimposition. In addition, the dataset was intentionally composed of radiographs with adequate diagnostic quality and without severe artifacts or major anatomical superimposition affecting the region of interest, allowing a more standardized evaluation of the models. Therefore, the findings may not fully reflect routine clinical conditions, where image quality and anatomical complexity are often more variable. Future studies using unselected real-world panoramic radiographs are needed to further assess the generalizability of these results. In addition, the models were evaluated under zero-shot conditions, without fine-tuning, supervised training, or additional clinical context, which may have influenced the observed diagnostic performance.

Another relevant aspect relates to the use of publicly available versions of the LLMs, whose internal parameters and updates are not fully controllable by researchers. Furthermore, the evaluation was performed using standardized image crops and a single experienced endodontic specialist as the reference standard. Although excellent intraexaminer reproducibility was observed, the use of a single examiner and panoramic radiographs may be associated with observer-dependent interpretation and the inherent limitations of this imaging modality for detecting small pulp calcifications. Additionally, because the cropped panoramic images did not include explicit orientation markers or broader anatomical context, some localization errors may have reflected difficulties in determining tooth position or laterality rather than failure to identify the pulp stone itself. To partially address this issue, localization performance was evaluated using both exact tooth identification and a more flexible dental group identification criterion. Moreover, repeated evaluations of the same image crops were necessary to assess response consistency, one of the primary objectives of the study. Therefore, the reported diagnostic metrics reflect model performance across multiple independent inference sessions rather than exclusively across unique image observations, which should be considered when interpreting the results.

Despite these limitations, the present study highlights both the potential and the challenges of multimodal LLMs in detecting pulp stones on panoramic radiographs, providing support for the future development of more specific, reproducible, and clinically applicable systems for the interpretation of dental images.

## Conclusion

ChatGPT and Gemini multimodal models demonstrated limited capability in detecting pulp stones on panoramic radiographs of posterior teeth, exhibiting distinct diagnostic profiles. ChatGPT showed higher specificity and greater intra-model stability, whereas Gemini demonstrated higher sensitivity and better performance in the anatomical identification of affected teeth. Nevertheless, both models presented limitations related to overall agreement, reproducibility, and diagnostic accuracy. Therefore, the findings suggest that, although multimodal LLMs may eventually contribute as auxiliary tools in the interpretation of dental images, their current performance still does not support consistent clinical applicability for the detection of pulp stones on panoramic radiographs.

## Supplementary Information

Supplementary Material 1.

## Data Availability

The datasets generated and/or analysed during the current study are available from the corresponding author on reasonable request.

## Abbreviations

AI: Artificial Intelligence
CI: Confidence Interval
F1-score: Harmonic Mean of Precision and Recall
GEE: Generalized Estimating Equations
LLMs: Large Language Models
NPV: Negative Predictive Value
PPV: Positive Predictive Value
GPT: Generative Pre–trained Transformer
SD: Standard Deviation
95% CI: 95% Confidence Interval
